# Preoperative CT-based deep learning model for predicting overall survival in patients with high-grade serous ovarian cancer

**DOI:** 10.3389/fonc.2022.986089

**Published:** 2022-09-09

**Authors:** Yawen Zheng, Fang Wang, Wenxia Zhang, Yongmei Li, Bo Yang, Xingsheng Yang, Taotao Dong

**Affiliations:** ^1^ Department of Obstetrics and Gynecology, Qilu Hospital of Shandong University, Jinan, China; ^2^ Department of Radiology, Qilu Hospital of Shandong University, Jinan, China; ^3^ Operating room, Qilu Hospital of Shandong University, Jinan, China; ^4^ Department of Radiology, Qingzhou People’s Hospital, Qingzhou, China

**Keywords:** ovarian cancer, survival prediction, deep learning, personalized model, nomogram

## Abstract

**Purpose:**

High-grade serous ovarian cancer (HGSOC) is aggressive and has a high mortality rate. A Vit-based deep learning model was developed to predicting overall survival in HGSOC patients based on preoperative CT images.

**Methods:**

734 patients with HGSOC were retrospectively studied at Qilu Hospital of Shandong University with preoperative CT images and clinical information. The whole dataset was randomly split into training cohort (n = 550) and validation cohort (n = 184). A Vit-based deep learning model was built to output an independent prognostic risk score, afterward, a nomogram was then established for predicting overall survival.

**Results:**

Our Vit-based deep learning model showed promising results in predicting survival in the training cohort (AUC = 0.822) and the validation cohort (AUC = 0.823). The multivariate Cox regression analysis indicated that the image score was an independent prognostic factor in the training (HR = 9.03, 95% CI: 4.38, 18.65) and validation cohorts (HR = 9.59, 95% CI: 4.20, 21.92). Kaplan-Meier survival analysis indicates that the image score obtained from model yields promising prognostic significance to refine the risk stratification of patients with HGSOC, and the integrative nomogram achieved a C-index of 0.74 in the training cohort and 0.72 in the validation cohort.

**Conclusions:**

Our model provides a non-invasive, simple, and feasible method to predicting overall survival in patients with HGSOC based on preoperative CT images, which could help predicting the survival prognostication and may facilitate clinical decision making in the era of individualized and precision medicine.

## Introduction

In the gynecological field, with an estimated 184,799 deaths worldwide annually, ovarian cancer is one of the most common and deadliest tumors. Among them, high-grade serous carcinoma subtype (HGSOC) is the most aggressive form and accounts for the majority of mortality (1). Despite advances in HGSOC therapy, such as surgery, chemotherapy, targeted therapy and immunotherapy, the 5-year overall survival is still substantial, less than 50% (2). Currently, stratification of HGSOC risk is still based on the stage of International Federation of Gynecology and Obstetrics (FIGO) (3), but because of the spatial and temporal heterogeneity of tumors, clinical biomarkers provide only partial information (4).

Currently, with its capacity to visualize a cancer’s appearance at a macroscopic level noninvasively, medical imaging contains more prognostic information for the primary tumor (5). Growing evidence suggests that computed tomography (CT) contains more mineable high-dimensional data which could promote personalized care and survival prediction in cancer patients (6–8). Despite the advantages that CT could quantify tumor shape and texture information, the existing hand-crafted feature engineering is difficult to extract the full intrinsic characteristics and prone to human biases (4, 8).

In recent years, with the development of optimization techniques and the improvement in computing devices, deep learning is becoming a popular method in medical image analysis (9). Because of its unique ability to learn features from raw data, the intrinsic characteristics of images have been mined automatically, thus reducing information redundancy and aiding clinical decision (10, 11). With the Transformer architecture, the Vision Transformer (ViT) has been shown to model long-range dependency among pixels and demonstrated the state-of-the-art (SOTA) performance in image classification task (12, 13). The attention mechanism in ViT model allows integration of global information but not of the local information from the CT images, which could have a significant impact on classification tasks. Existing literature describes the use of the ViT model to predict medical targets, such as emphysema classification (14) and COVID-19 diagnosis (15).

In this study, we developed a Vit-based deep learning model to predicting overall survival in patients with HGSOC based on preoperative CT images. Instead of delineating precise tumor boundaries which often used in conventional radiomics methods, our model requires only a rectangle region of tumor, thus reducing the interobserver error and manual segmentation time. Moreover, integrating the prediction features learned from CT images, clinicopathological and hematological markers, we established a comprehensive nomogram aiming to provide a non-invasive individualized recurrence prediction model in HGSOC.

## Material and methods

### Patients

Ethical clearance of this retrospective study was obtained and the requirement for informed consent was waived. Preoperative CT images of 734 patients were collect from the Qilu Hospital of Shandong University. The whole dataset was randomly split into 75% for training cohort and 25% validation cohort, which were mutually exclusive. All patients were conducted every three months during the first three years after surgery, and every six months thereafter. The primary endpoint of this study was the occurrence of death, the median follow-up time was 35.6 months.

Our inclusion criteria of the data were as follows: (1) pathologically confirmed primary HGSOC; (2) primary debulking surgery was performed and clinical complete remission was achieved after treatment; and (3) available preoperative ultrasound Contrast-enhanced CT data. Our exclusion criteria were as follows: (1) incomplete clinical data (preoperative CA-125, age, FIGO stage, etc.) or survival data; and (2) unqualified CT images (e.g., motion artifacts).

### CT image

For all patients, contrast-enhanced CT scanning was acquired at diagnosis. All the patients were examined using a multi-detector row spiral CT (MDCT) scanner (Philips Brilliance iCT) with the following scanning parameters: tube voltage, 120 kVp; tube current, automatic; beam pitch, 1; reconstruction thickness, 1mm; reconstruction interval, 1 mm. Contrast-enhanced venous phase CT scan was used in this study. The contrast agent used was as follows: Ultravist 300, Bayer, Germany; contrast medium dose, weight (kg) ×1.2 mL; injection rate, 3 mL/sec. Scanning began 70 s after injection using a power injector.

CT examinations in this study were strictly performed in accordance with the principle of “ALARA” (i.e. as low as reasonable achievable). During the period of examination, patient was scanned in suspended respiration. The scanning area was from the symphysis pubis to the diaphragm. A radiologist (10+ years’ experience, Dr. Fang Wang) manually selected a rectangle region of interest (ROI) containing the entire tumor in all CT slices form 734 patients. If multiple tumor areas are observed in one CT slice, multiple ROIs will be selected. At last, 16517 tumor images were got for the deep learning model training.

### Development of the deep learning model

**Figure 1:**
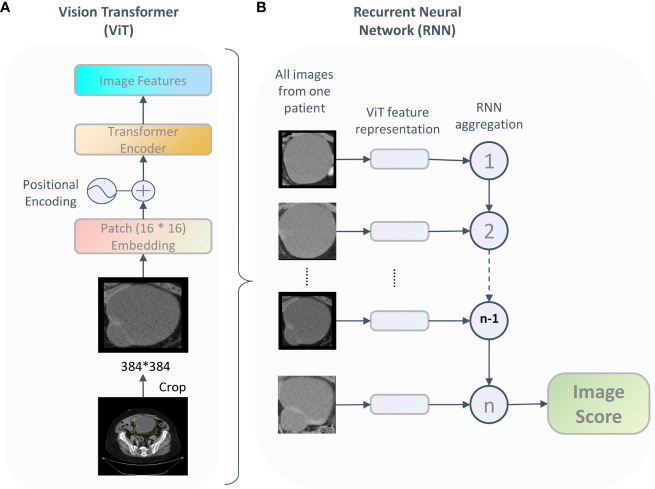
The framework of the proposed Vit-based model. For each patient, being fed tumor images, the deep learning model output an image score which represent the patient’s survival probability. This framework includes two parts: the ViT part **(A)** learned features and the RNN part **(B)** integrated the feature representation for each patient and reported the final image score.

We developed a Vit-based deep learning model to predicting overall survival in patients with HGSOC based on preoperative CT images, as shown in **Figure 1**. The model included two parts: the Vit part and the RNN part. The Vit part comprises a linear embedding layer, a transformer encoder block, and a feature-learning layer. In this part, after scaling to the same size (384 * 384 pixels), all tumor images were fed into the Vit (13), resulting in a semantically rich feature representation. Then, a recurrent neural network (RNN) was used to integrate the feature representation for each patient and reported the final image score which indicating the individual death risk. This image score was used for overall survival prediction and to stratify patients into different risk groups.

The deep learning model was implemented based on the PyTorch (vision 1.10.2) and Python 3.8. A variety of data augmentation techniques were used to increase the variability of training data during pre-training, including random flipping, rotation, and translation. Initializing with ImageNet pretrained weights, we trained the model with 70% of the data in the training cohort and use the rest of the dataset as the test set to get the best-performing model with a batch size of 16. Finally, the model was evaluated on the validation cohort. The patch size is 16 * 16, minimization of the loss was achieved *via* stochastic gradient descent (SGD) using the Adam optimizer and learning rate 0.0001. We used 128 dimensional vectors for the state representation of the recurrent unit and the recurrent steps was 100.

### Overall survival analysis

To evaluate the prognostic value of the image score, we built a predictive model involving multimodal features and parameters such as image score, age, tumor diameter, FIGO stage, preoperative CA-125, and tumor location based on the resulting coefficients from the multivariate Cox analysis in the training cohort, and further constructed a nomogram. Then, calibration curves were drawn to evaluate and validate the application ability of the nomogram performance. Besides, the 3- and 5-year survival probability were predicted by the nomogram and both predicted probabilities and the observed probabilities were illustrated by calibration curves.

### Statistical analysis

Clinical characteristics were compared between the training set and the validation set by using the Mann-Whitney U test, x^2^ test, or chi-square test, as appropriate. The significance of correlation between two covariates were assessed by Pearson correlation test. Based on quartiles of image scores, patients were categorized into four groups for survival analysis. Survival curves for overall survival were estimated using the Kaplan–Meier method, and comparisons of statistical significance were performed with the stratified log-rank test within each group. Multivariable analyses were performed using the Cox proportional hazards model. Harrell’s concordance-index (C-Index) was used to measure the concordance between the DL-predicted death risk and the actual survival time. The receiver operating characteristic curve (AUC) was used to measure the discriminatory power of the model to predict survival.

All the statistical analyses were performed by using R software (version 3.6.3; http://www.R-project.org) with packages rms, survival, survminer, and Hmisc. A P value of less than 0.05 was considered to indicate statistical significance.

## Results

### Characteristics of patients

**Table 1** summarized the clinical characteristics in training and validation cohorts. The median follow-up interval was 35.6 months (interquartile range, 20.7-58.6 months), and death was observed in 25.89% (190 of 734). No significant difference was observed between the training and validation cohorts with regard to age, tumor diameter, FIGO stage, preoperative CA-125, tumor location, and vital status.

**Table 1:** Clinical characteristics of patients in training and validation cohorts.

	Total N = 734	Training cohort N = 550	Validation cohort N = 184	p-value
Age at diagnosis, mean (SD), y	52.36 (15.18)	52.59 (15.24)	51.75 (15.08)	0.54
Follow-up time, median (IQR), m	35.6 (20.7, 58.6)	35.0 (20.0, 57.4)	36.9 (21.8, 61.1)	
Tumor diameter, mean (SD), mm	10.10 (6.04)	10.23 (6.10)	9.73 (5.87)	0.36
CA-125, mean (SD) , U/ml	1019.3 (1381.1)	1042.7 (1437.9)	949.5 (1196.2)	0.90
Tumor location, No. (%)				
Unilateral	307 (41.83)	220 (40.00)	87 (47.28)	0.10
Bilateral	427 (58.17)	330 (60.00)	97 (52.72)	
FIGO stage, No. (%)				
I	209 (28.47)	155 (28.18)	54 (29.35)	0.94
II	77 (10.49)	59 (10.73)	18 (9.78)	
III	386 (52.59)	291 (52.91)	95 (51.63)	
IV	62 (8.44)	45 (8.18)	17 (9.24)	
Vital status , No. (%)				
Alive	544 (74.11)	412 (74.91)	132 (71.74)	0.45
Dead	190 (25.89)	138 (25.09)	52 (28.26)	

**Figure 2:**
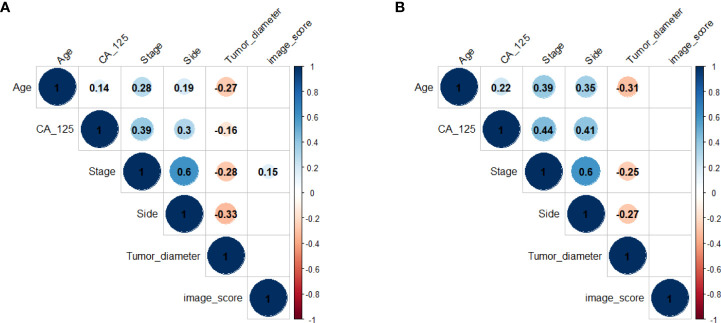
Correlation matrix of clinical characteristics and the image score in training cohort **(A)** and validation cohort **(B)**. Values in this figure indicated the correlation coefficient of two corresponding variables. The colour and the size of the circles represent the strength of the correlation. Lack of color means no correlation.

The correlations between all clinical characteristics and the image score got from the deep learning model in training cohort and validation cohort are presented on the scatterplot matrix shown in **Figure 2**. Notably, the correlation matrix showed that the image score was not correlated with all clinical characteristics in validation cohort and was only weakly correlated with stage in training cohort. The results suggested that the image score obtained from our deep learning model was an independent prognostic factor for the survival time of patients with HGSOC.

### Performance of the proposed deep learning model

A Vit-based deep learning model using preoperative CT images was proposed in this study to predict overall survival in patients with HGSOC. The model framework is described in the Materials and Methods and shown in **Figure 1**.

**Figure 3:**
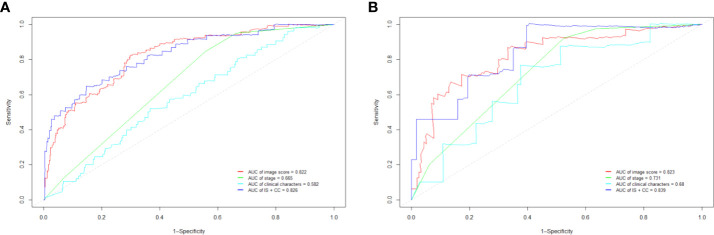
The receiver operating characteristic curve (ROC) in training cohort **(A)** and validation cohort **(B)**. IS, image score; CC, clinical characters.

Our Vit-based deep learning model showed promising results in predicting survival, with an AUC of 0.822 (95% CI: 0.804–0.858) in the training cohort and 0.823 (95% CI: 0.795–0.862) in the validation cohort (**Figure 3A**). The sensitivity of the model was 85.2% in the training cohort and 83.7% in the validation cohort, while specificity was 72.4% in the training cohort and 69.5% in the validation cohort. According to the ROC curve, this model has a prognostic value exceeding that of FIGO stage and all clinical characters (**Figure 3**). When we combined image score and clinical characters together, we found that the ROC score appeared similar to that of image score alone.

### Multivariable Cox regression analysis

As we mentioned above, the correlation matrix suggested that the image score obtained from our model was an independent prognostic factor for OS, we next performed the univariate and multivariate cox regression analyses to further characterize the association between the image score and survival (**Table 2**). The multivariate Cox regression analysis indicated that the image score and FIGO stage were independent prognostic factors in the training and validation cohorts. The multivariable-adjusted HRs of the image score were 9.03 (95% CI: 4.38, 18.65; p < 0.001) in training cohort and 9.59 (95% CI: 4.20, 21.92; p < 0.001) in validation cohort.

**Table 2:** Univariable and Multivariable Analyses of Overall Survival in training and validation cohorts.

Univariable Analysis				
	Training cohort		Validation cohort	
	HR (95%CI)	p-value	HR (95%CI)	p-value
Age at diagnosis	1.03 (1.01-1.04)	< 0.001	1.02 (1.00-1.03)	0.13
Tumor diameter	0.97 (0.94-1.0)	0.04	0.97 (0.93-1.02)	0.289
CA-125	1 (1-1)	0.04	1 (1-1)	< 0.001
Side				
Unilateral	1.0 (referent)	referent	1.0 (referent)	referent
Bilateral	1.65 (1.14-2.38)	0.008	2.37 (1.28-4.38)	0.006
FIGO stage				
I	1.0 (referent)	referent	1.0 (referent)	referent
II	4.05 (2.06-7.97)	< 0.001	2.86 (1.35-6.09)	0.006
III	5.34 (3.13-9.12)	< 0.001	4.34 (2.48-7.60)	< 0.001
IV	6.54 (3.45-12.42)	< 0.001	5.25 (2.63-10.48)	< 0.001
Image_score	7.8 (3.83-15.88)	< 0.001	6.84 (3.07-15.27)	< 0.001
**Multivariable Analysis**				
	**Training cohort**		**Validation cohort**	
	**HR (95%CI)**	**p-value**	**HR (95%CI)**	**p-value**
Age at diagnosis	1.01 (1.00-1.03)	0.008	1.02 (0.99-1.05)	0.12
Tumor diameter	1.00 (0.96-1.03)	0.85	0.99 (0.94-1.04)	0.64
CA-125	1.00 (1.00-1.00)	0.86	1.00 (1.00-1.00)	0.02
Side, No.(%)				
Unilateral	1.0 (referent)	referent	1.0 (referent)	referent
Bilateral	0.84 (0.06-1.27)	0.41	0.91 (0.45-1.81)	0.78
FIGO stage, No.(%)				
I	1.0 (referent)	referent	1.0 (referent)	referent
II	3.50 (1.75-7.00)	< 0.001	1.23 (0.24-6.29)	0.81
III	5.52 (3.03-10.04)	< 0.001	3.38 (1.30-8.83)	0.01
IV	5.60 (2.72-11.50)	< 0.001	4.65 (1.38-15.73)	0.01
Image_score	9.03 (4.38-18.65)	< 0.001	9.59 (4.20-21.92)	< 0.001

**Figure 4:**
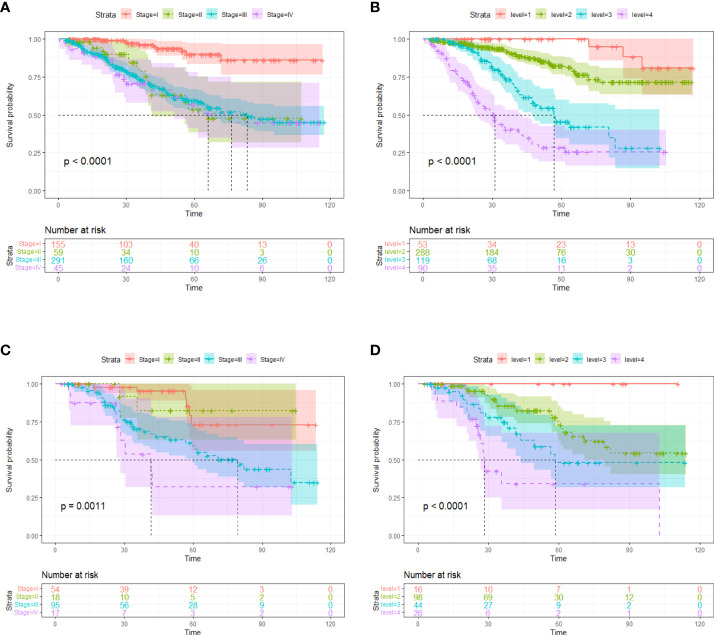
Kaplan-Meier survival curves according to tumor stages in training cohort **(A)** and validation cohort **(C)**. Kaplan-Meier survival curves based on risk stratification according to image score in training cohort **(B)** and validation cohort **(D)**. The shadow indicates the 95% confidence interval.

Though FIGO stage is an acknowledged risk factor for HGSOC survival, the Kaplan–Meier survival analysis suggested that only patients with stage I disease had significantly better OS compared to patients with stage II/III/IV disease (p < 0.001), the survival did not differ significantly between stage II, III and IV patient subgroups (**Figure 4**). Therefore, considering that FIGO stage could not stratified patients exactly, we categorized patients into four groups based on the cut-off values of the image score. Significant discrimination between the patient survival of the four groups was observed in the two cohorts (all p < 0.05, log-rank test) (**Figure 4**).

### Nomogram construction and validation

**Figure 5:**
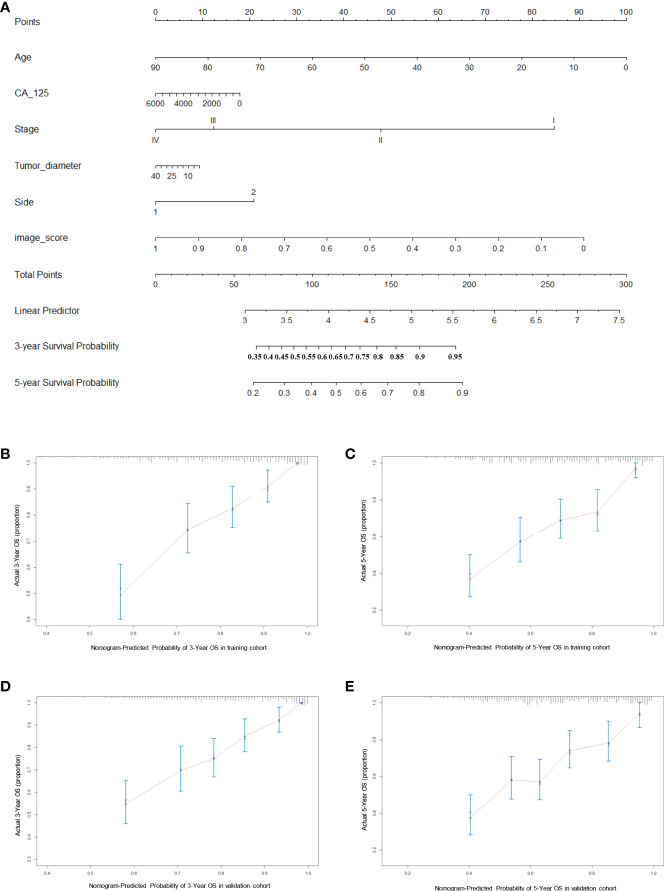
Generation and evaluation of nomogram. **(A)** A constructed nomogram for prognostic prediction of 3-year and 5-year overall survival for patients with HGSOC. **(B, C)** Calibration curves of 3-year and 5-year OS for HGSOC patients in the training cohort. **(D, E)** Calibration curves of 3-year and 5-year OS for HGSOC patients in the validation cohort. Dash line represents the ideal agreement, the red dots are calculated by bootstrapping.

Finally, we constructed a nomogram for HGSOC survival prediction on the basis of the selected clinical characters and model-based image score (**Figure 5A**). By drawing a vertical line down to the axis labeled points, each covariate in the model was assigned a score. A 3-year or 5-year survival probability can be calculated by summing the total score and placing it on the total points scale. The C-index value was 0.74 in the training cohort and 0.72 in the validation cohort. Further, the calibration curves demonstrated high consistency between the nomogram-predicted 3-year and 5-year survival probabilities and the actual outcome in both the training and validation cohorts (**Figures 5B–E**). Our above results revealed that the nomogram for had a high discriminative and calibrating power.

## Discussion

Our work proposed a novel and innovative deep learning framework for prognosis and prediction in HGSOC based on preoperative CT images. Combined Vision Transformer with RNNs, our model extracted an image score from preoperative CT images for each patient, which was then shown to be an independent prognostic factor for HGSOC (AUC is 0.822 in the training cohort and 0.823 in the validation cohort) and had prognostic value above other clinical characters. Then, incorporated clinical characters and model-based image score, a concise nomogram was performed for survival prediction and intended to serve as a practical guide to clinicians when recommending an appropriate management strategy for HGSOC patients.

In recent years, radiomics has grown rapidly as a medical research field due to the combination of radiographic images and data about clinical outcomes. In earlier radiomics studies, medical images are assessed visually by trained physicians for the purpose of detecting, characterizing, and monitoring diseases, which often relies on education and experience and can be subjective at times. Deep learning, however, excels at identifying complex patterns and can provide automated quantitative assessments in contrast to qualitative reasoning (16). More meaningfully, the feature representations automatically learned by deep learning techniques hinting at the substantial clinical relevance of many of these radiographic features (17). Preoperative CT examination is very necessary for patients with solid tumors and it may contain multiple anatomic and nonanatomic elements which may enhance the capacity to prognosticate (18, 19). In ovarian cancer, preoperative CT is an important means of staging and treatment decision (20, 21), however, the prognostic information provided by manual observation is limited, we believe that through deep learning, more information about prognosis could be directly extracted and could constitute the prognostic index for patients with ovarian cancer. Several previous studies reported the efficacy of deep learning for the survival prediction of cancer on radiology images (7, 22–24), but most of them have used traditional CNNs. Wang et al. constructed a non-invasive recurrence prediction model based on CNN (8). Avesani et al. used a CNN as feature extractor to predict progression free survival and BRCA mutational status (25). Comprising 44,732 slides from 15,187 patients, Gabriele et al. developed a deep learning frame-work that combined CNNs with RNNs to diagnose prostate cancer, the semantically rich tile-level feature representations resulted from CNNs were then used in a RNN to integrate the information across the whole slide and report the final classification result (26). The AUC of this model was above 0.98 and its clinical application would allow pathologists to exclude 65–75% of slides while retaining 100% sensitivity. Although in the field of medical image analysis, CNNs have been widely adopted, they have inherent limitations. CNN is good at focusing on extraction of local information, but this means the receptive field is limited and the global feature is hard to be captured.

In contrast, mainly based on self-attention mechanisms, the primary advantage of Transformer is its global receptive field and focus on the aggregation of global information. In the past few years, Transformer have been dominant in the natural language processing field and have been used in speech recognition (27), machine translation (28), and language modeling (29). More recently, to overcome these limitations of CNN in computer vision problems, equipped with the Transformer architecture, Vision Transformer (ViT) was proposed to model long-range dependency among pixels through the self-attention mechanism (12), and has been demonstrated the state-of-the-art (SOTA) performance in a variety of vision tasks including object detection (30), classification (13), segmentation (31), and so on. At present, in the field of cancer, there are several researches using ViT for classify tasks (32–34) and cancer region detection and segmentation tasks (35). As far as we know, our study is the first to attempt to apply ViT for the survival prediction of HGSOC. Our proposed ViT-based model integrates the advantages of ViT and RNN, enabling the model to have overwhelming effect on survival prediction.

Although our model performed well, there are also several limitations. First, the CT images to construct the model were collected from only one manufacture, different CT scanners may lead to distinct image features. Second, since we still require manual tumor annotation (although only bounding box) on CT images, our model is not fully automated. Third, we lacked an additional cohort for external validation. Finally, we only focus on high-grade serous carcinoma subtype, further validation studies are warranted for other ovarian cancer subtypes. Hence, in future, a more general and more robust model which was trained by more data should be considered. And our research team is currently collecting more data from other medical centers to further validate and improve the current model. In addition to preoperative CT images and clinical information, whole slide images (WSIs) of hematoxylin and eosin-stained postoperative pathological slides were also collected to develop the multimodal model.

In conclusion, this study shows a new deep learning model which could output an independent prognostic risk score for predicting survival in patients with HGSOC through their preoperative CT images. Combined with other clinical characters, the score was used to construct a simple, yet not trivial nomogram, which would have potential as a useful tool in creating optimal individualized therapeutic approaches for HGSOC patients. Our study could help predicting the survival prognostication for HGSOC patients and may facilitate clinical decision making in the era of individualized and precision medicine.

## Data availability statement

The original contributions presented in the study are included in the article/supplementary material. Further inquiries can be directed to the corresponding authors.

## Ethics statement

The studies involving human participants were reviewed and approved by Institutional Ethics committee of Qilu Hospital. The patients/participants provided their written informed consent to participate in this study. Written informed consent was obtained from the individual(s) for the publication of any potentially identifiable images or data included in this article.

## Author contributions

YZ: Conceptualization, methodology, and writing (original draft). FW: Conceptualization, image processing and methodology. WZ: Formal analysis, data curation and writing (review and editing). YL: Formal analysis and data curation. BY: Image processing and formal analysis. XY: Conceptualization, and supervision. TD: Conceptualization, project administration and funding acquisition. All authors contributed to the article and approved the submitted version.

## Funding

This study was funded by Innovation and Development Joint Funds of Natural Science Foundation of Shandong Province (ZR2021LZL009).

## Conflict of interest

The authors declare that the research was conducted in the absence of any commercial or financial relationships that could be construed as a potential conflict of interest.

## Publisher’s note

All claims expressed in this article are solely those of the authors and do not necessarily represent those of their affiliated organizations, or those of the publisher, the editors and the reviewers. Any product that may be evaluated in this article, or claim that may be made by its manufacturer, is not guaranteed or endorsed by the publisher.
